# Correlation between osteoprotegerin and coronary artery calcification in diabetic subjects: a systematic review of observational studies

**DOI:** 10.1186/s12872-023-03123-z

**Published:** 2023-02-21

**Authors:** Fatemeh Vazirian, Masoumeh Sadeghi, Dongdong Wang, Reza Javidi Dashtbayaz, Arash Gholoobi, Sara Samadi, Amir Hooshang Mohammadpour

**Affiliations:** 1 School of Pharmacy, Mashhad University of Medical Sciences, Mashhad, Iran; 2 Department of Epidemiology, Faculty of Health, Mashhad University of Medical Sciences, Mashhad, Iran; 3 Department of Medicine, Centre for Metabolism, Obesity and Diabetes Research, McMaster University, Hamilton, ON Canada; 4 Department of Cardiovascular Diseases, Faculty of Medicine, Mashhad University of Medical Sciences, Mashhad, Iran; 5 Department of Clinical Pharmacy, School of Pharmacy, Mashhad University of Medical Sciences, Mashhad, Iran; 6 Pharmaceutical Research Center, Pharmaceutical Technology Institute, Mashhad University of Medical Sciences, Mashhad, Iran

**Keywords:** OPG, Bone markers, Atherosclerosis, Diabetes, Coronary artery calcium score, Cardiovascular disease

## Abstract

Coronary artery calcification (CAC) is one of the critical cardiovascular complications that lead to elevated morbidity and mortality among patients with type 2 diabetes (T2M). The association between osteoprotegerin (OPG) and CAC could potentially provide a reasonable chance for preventive therapy in type 2 diabetic patients and benefit the rate of mortality. Since measurement of CAC score is relatively expensive and requires radiation exposure, the current systematic review aims to provide clinical evidence for evaluating the prognostic role of OPG in determining CAC risk among subjects with T2M. Web of Science, PubMed, Embase, and Scopus, were investigated until July 2022. We assessed human studies investigating the association of OPG with CAC in type 2 diabetic patients. Quality assessment was performed by Newcastle–Ottawa quality assessment scales (NOS). Out of 459 records, 7 studies remained eligible to be included. Observational studies that provided odds ratio (OR) estimates with 95% confidence intervals (CIs) for the association between OPG and the risk of CAC were analyzed by random-effects model. In order to provide a visual summary of our findings, the estimation of pooled OR from cross-sectional studies was reported as 2.86 [95% CI 1.49–5.49], which is consistent with the findings of the cohort study. Results revealed that the association between OPG and CAC was significant among diabetic patients. OPG is hypothesized to be a potential marker in predicting the presence of high coronary calcium score among subjects with T2M that could be recognized as a novel target for further pharmacological investigations.

**Supplementary Information:**

The online version contains supplementary material available at 10.1186/s12872-023-03123-z.

## Introduction

Type 2 diabetes (T2M) is responsible for vascular aging and is associated with prolonged complications and cardiovascular disease (CVD) mortality and morbidity [1, 2]. The accumulative evidence suggests that hyperglycemia is a key contributing factor in CVD. As seen in a recent population-based study with 13-year follow-ups, HbA1c and higher blood glucose were strongly associated with the CVD incidence among type 2 diabetic patients [3]. Accordingly, a higher glucose level in circulation would facilitate the production of reactive oxygen metabolites, which decreases nitric oxide (NO) bioavailability leading to arterial stiffness and endothelium dysfunction [4]. Moreover, oxidative stress plays an important role in the development of coronary artery calcification (CAC) by inducing the transdifferentiation of endothelium cells into osteoblast-like cells, leading to increased levels of osteoprotegerin (OPG) [5, 6]. OPG contributes to bone mineralization by inhibiting RANKL receptors and osteoclasts` recruitment [7–9]. The accumulative results from both in vitro and in vivo studies demonstrated that the expression of OPG is increased following hyperglycemia [10, 11].

Calcification in the arteries will promote inflammatory cytokines migration and interfere with the blood flow by damaging endothelial arteries. CAC contributes to plaque formation and atherosclerosis [12]. Given the predictive role of CAC in identifying subclinical CVD patients among all races in both men and women at any age [13, 14], evaluating the association between OPG and CAC following hyperglycemia could highlight the potential targeting role of OPG in the early diagnosis of CVD in type 2 diabetic patients.

There is controversial evidence considering the significant association of OPG with CAC in T2M. In a prospective study recruiting type 2 diabetic subjects with a mean follow-up of 18 months, OPG was identified as a strong predictor of CAC scores [15]. However, OPG was demonstrated not to be associated with CAC burden in a cross-sectional study with type 2 diabetic individuals [16]. Since T2M enhances the burden of atherosclerotic cardiovascular diseases, the current study aims to systematically evaluate the association between levels of OPG and CAC in type 2 diabetic patients that provide potential clinical evidence for identifying OPG as a diagnostic marker in CAC stratification.

### Hyperglycemia and OPG: mechanism of action

**Fig. 1:**
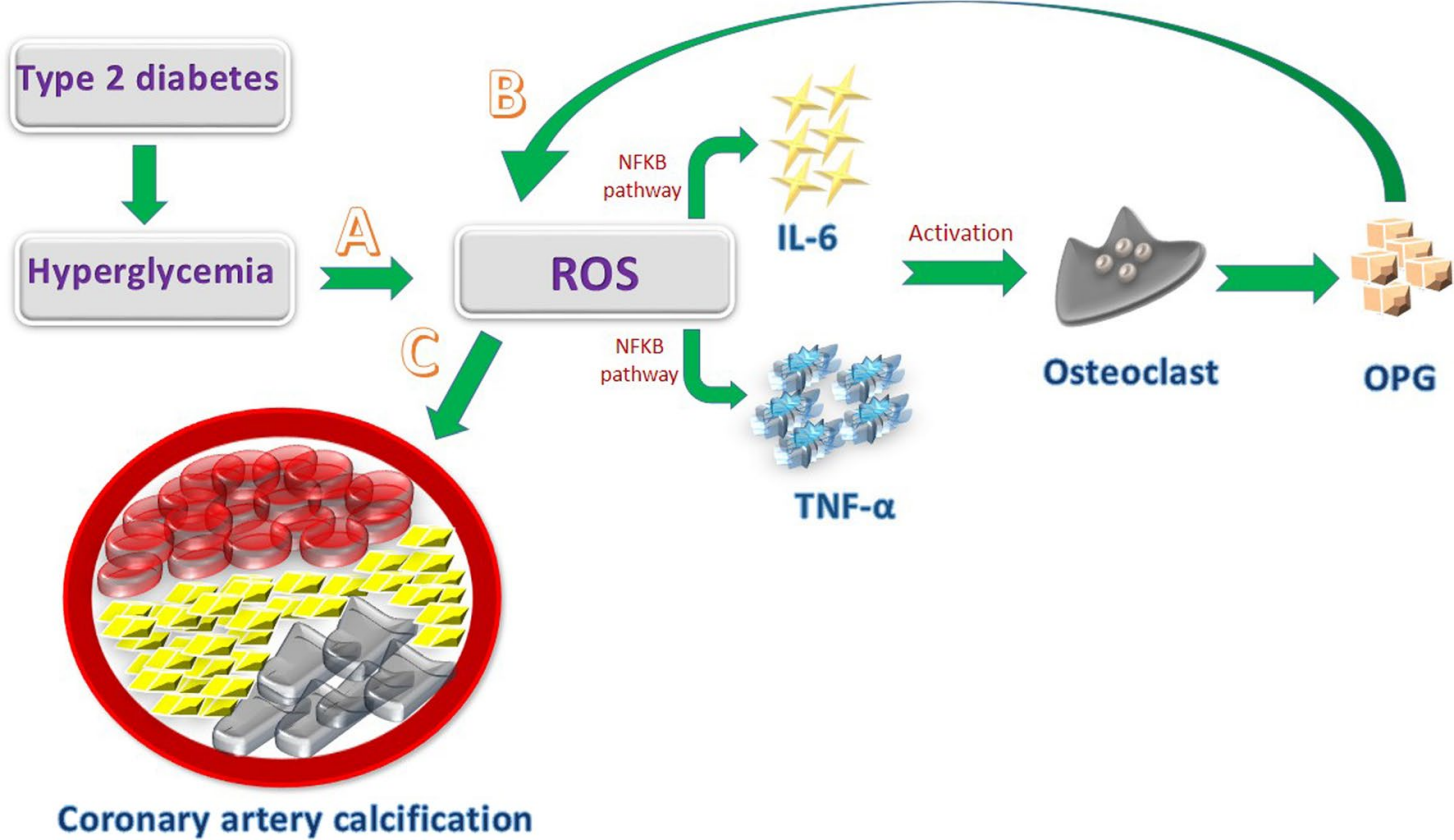
Mechanistic approach towards the role of hyperglycemia and OPG in CAC. **A** Hyperglycemia is responsible for the accumulation of excessive ROS level that reflects oxidative stress. Following the ROS stimulation, NF-κB signaling pathway is triggered and the expression of inflammatory cytokines including TNF-α and IL-6, is increased. TNF-α and IL-6 are responsible for promoting osteoclast differentiation and the expression of OPG is increased during the osteoclastogenesis phase. **B** The elevated level of OPG can induce ROS accumulation in cells and extensive oxidative stress will occur. **C** ROS plays a crucial role in artery endothelial damage through various pathways leading to CAC occurrence and atherosclerosis in T2M patients

Hyperglycemia is the bridge linking T2M and oxidative stress, leading to the accumulation of excessive reactive oxygen species (ROS) in the circulation. Hyperglycemia also accelerates mitochondrial dysfunction and promotes ROS production [17]. ROS is a contributing factor for intracellular pathway modifications including the NF-κB signaling pathway, which plays an essential role in regulating the synthesis of inflammatory cytokines. Following the activation of the NF-Κb pathway, the transcription of cytokines including TNF-α, IL-6, IL-2, and IL-8 and adhesion molecules such as ICAM-1 and VCAM-1is induced, and the inflammatory cascade is commenced in the body [18, 19] (Fig. 1). Owing to the elevated levels of TNF-α and IL-6, the extensive activity of osteoclasts is stimulated and leads to the induction of osteoclastogenesis [20]. As seen in a study evaluating the level of OPG in a rat model, the expression of OPG is increased during the late phase of osteoclastogenesis to inhibit the osteoclast activity and prevent bone resorption in the body [6, 21]. Moreover, the in vitro study indicated that OPG expression is responsible for osteoclast enlargement and regulates osteoclast differentiation [6]. Meanwhile, the elevated level of OPG is an associated modulator of ROS activation and induces oxidative stress in cells [22]. Given the notable roles of ROS in atherosclerosis, several mechanisms contributing to vascular plaque formation are proposed: (1) ROS promotes lipid oxidation and endothelial damage will be the consequence [23]. (2) ROS induces an osteochondrogenic trans-differentiation pathway and vascular smooth muscle cells (VSMCs) are switched to osteoblast leading to mineral calcification in the vascular endothelium [5]. Therefore, the oxidative stress induced by hyperglycemia can increase the level of OPG in diabetic patients and the process of CAC is stimulated in these subjects. Since hyperglycemia has a direct role in activation of oxidative stress pathways and increases the expression of OPG, the pharmacological targets that can reduce the level of blood glucose will be of interest to reduce hyperglycemia in diabetic patients and inhibit one of the first steps in the trans-differentiation pathway of VSMCs. As seen in a recent study, sodium glucose transporter 2 (SGLT2) inhibitors indicated cardio-protective effects in patients with cardiovascular symptoms by reducing the plasma level of glucose and decreasing the production of inflammatory factors such as interleukin 6 (IL-6) [24]. Regarding the cardio-effective impacts of SGLT2 inhibitors, a recent study evaluating type 2 diabetic patients with acute myocardial infarction indicated that administering SGLT2 inhibitors was associated with lower incidence of new-onset cardiac arrhythmias in patients (OR 0.35; 95% CI 0.14–0.86; *p* = 0.022) [25]. Therefore, using novel targets that can decrease hyperglycemia can further highlight their protective impacts on the cardiovascular events.

## Methods

The systematic review protocol was developed based on the Preferred Reporting Items for Systematic Review and Meta-Analysis (PRISMA) 2020 guidance [26] (Additional file 1: Appendix 1).

### Databases and search strategy

In this review, four databases, including Scopus, Embase, PubMed, and Web of Science, were investigated until the last week of July 2022. The PECO protocol was designed in three parts: T2M as the population, OPG level as exposure, and CAC as the outcome. We included the studies evaluated CAC as a primary or secondary outcome. Our search was conducted with the Mesh terms of osteoprotegerin, diabetes mellitus type 2, coronary artery disease, atherosclerosis, myocardial Ischemia, myocardial Infarction. The Emtree words used in our search were non insulin dependent diabetes mellitus, coronary artery disease, arteriosclerosis, atherosclerosis, heart muscle ischemia, heart infarction, ischemic heart disease, osteoprotegerin, and coronary artery calcification. Search strategies are available as additional files (Additional file 1: Appendix 2). Furthermore, all of references were investigated in related articles to find any overlooked articles.

### Eligibility criteria and study selection

The two investigators reviewed the titles and abstracts of retrieved records, and then full-text of records were searched for those that were supposed to meet our inclusion criteria. Moreover, we emailed twice for papers where full text was not available and in case of not receiving any answer, the article was excluded. To resolve any disagreement, a third author was asked to develop a confident result. We included observational studies, including case–control, cohort, and cross-sectional studies without language or time restrictions. Human studies that assessed the association of OPG with coronary artery calcium score in patients with T2M were evaluated. We excluded technical reports, conference papers, case reports, animal studies, and review articles.

### Data abstraction and quality assessments

Data was extracted independently by two reviewers and was sorted by the first author, year, country, age, population, number of participants, odds ratio; OR with their confidence intervals [CI]), the outcome of studies, and results. Newcastle–Ottawa quality assessment scales (NOS) were utilized for the quality assessment of the studies [27].

### Statistical analysis

All statistical analyses were carried out using Stata version 11.2 software (Stata Corp., College Station, TX, USA). Due to the high heterogeneity in methodological and study design, meta-analysis could not be performed by this study. We also provide a visual summary of our findings. A prospective cohort, one case control and two cross-sectional studies that were determined the relationship between CAC and OPG and also reported OR and 95% CI as measure of the association were included in our analysis. Using random effects meta-analysis, the heterogeneity of the study populations was examined and a forest plot was also created to determine the multivariable adjusted OR and corresponding 95% CI for visual inspection across the studies. Logarithm of the OR and its standard error was used in our analysis. Der-Simonian and Laird method was applied to calculate the estimation of pooled OR with its corresponding 95% CI. Heterogeneity between the studies was determined using the I^2^ statistic (I^2^ = 0% shows negligible heterogeneity and I^2^ ≥ 50% shows the substantial heterogeneity) which refers to the percentage of total variation that is true between-study heterogeneity. All statistical tests were two-tailed and significance level was considered less than 0.05 for all, except heterogeneity test.

## Results

### Results of the literature search

**Fig. 2:**
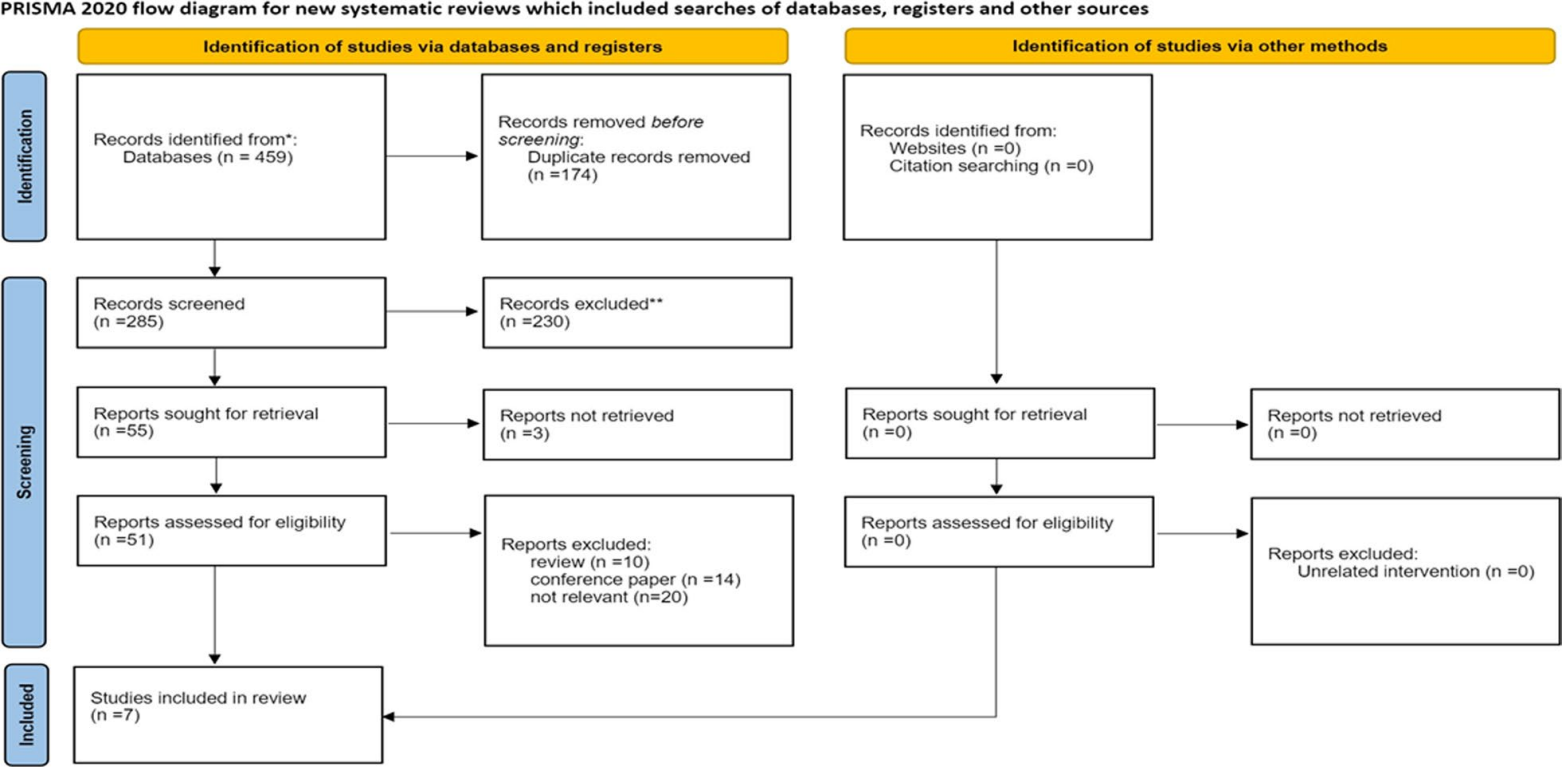
Flowchart of the study. *Consider, if feasible to do so, reporting the number of records identified from each database or register searched (rather than the total number across all databased /registers). ** If automation tools were used, indicate how many records were excluded by a human and how many were excluded by automation tools. From package MJ, McKenzie JE, Bossuyt PM, Boutron I, Hoffmann TC, Mulrow CD et al. The PRISMA 2020 statement: an updated guideline for reporting systematic reviews. BMJ 2021:372:n71. 10.1136/bmj.n71. For more information , visit http://www.prisma-statement.org/

Our systematic review retrieved 459 records from four databases which 174 duplicate studies were removed, and after assessing the eligibility criteria, seven records remained during the selection process (Fig. 2).

### General characteristics of included studies

In the eligible studies, both men and women were investigated. Three were conducted in Asia, one in the United States, and three in Europe among the included studies. Moreover, three studies recruited asymptomatic diabetic patients. The majority of the included studies were cross-sectional, and the mean age of all study participants was higher than fifty years old. The level of OPG in plasma was measured in four studies, while three studies reported the concentration of OPG in serum and all the studies used ELISA for measuring OPG. CAC was investigated as the outcome in all the included studies and cardiovascular events were also evaluated in two studies. The detailed characteristics of the studies are provided in Table 1. In addition, data pertinent to the relationships between OPG and traditional CVD risk factors were also presented in Table 2. In majority of the studies, age, fasting blood sugar, and duration of diabetes showed a positive correlation with serum OPG level. Considering the risk of bias assessment, most articles were ranked as good quality (Additional file 1: Appendix 3).

**Table 1:** Characteristics of the studies

References	Country	Age (Year)	Population	Type of study/n	Medication (n)	OPG (pmol/L)	OR [95% CI]	Adjustment variables	Result	Outcome	Quality
Anand [15]	UK	52.7	Asymptomatic T2DM Free of CVD symptoms	Prospective cohort/510	Antihypertensive (380), lipid-lowering (318), Insulin only (57), Insulin + oral agent (51)	Plasma 6.82	OR 2.84 [2.2–3.67], *p* < 0.01	Age, gender, hypertension, ethnicity, duration of diabetes, and statin use	OPG levels strongly associated with CAC > 10 scores	CAC-CAD	Good
Ishiyama [16]	Japan	60.85	T2DM Free of CAD symptoms	Cross-sectional/168	Diabetics: diet/OHD/insulin(18/102/48), Statins (51), ACEI or ARB (58)	Plasma 1.45	NR		OPG was not a significant independent determinant of CAC	CAC	Good
Jung [31]	Korea	57.2 ± 11.2	Asymptomatic T2DM patientFree of CAD symptoms	Cross-sectional/110	Statins (65), OHD only (77), OHA and Insulin (19), Insulin only (2). Use of statin was an independent variable for CAC (β = -0.315, p = 0.03)	Serum 12.5	NR		CAC and serum OPG levels were significantly correlated with each other	CAC	Good
Maser [28]	USA	62.5	T2DM Free of CVD symptoms	Cross-sectional/50	NR	Serum 5.5	OR 3.324 [1.321–8.359], *p* = 0.011	Duration of diabetes, Age, Systolic blood pressure, Gender, BMI, HOMA-IR, Leptin, HbA1c, Adiponectin	OPG is a useful serum biomarker for identifying subjects at increased risk of arterial calcification (CAC score ≥ 10)	CAC	Moderate
Godsland [30]	UK	67.4	12 T2DM patients with cardiovascular events and 12 T2DM patients free of CVD symptoms	Nested case–control/24	Drug use in high and low CACS groups was similar	Plasma 9.3	NR		OPG OPG differed at borderline significance between two groups of high and low CAC score	CAC	Moderate
Reinhard [29]	Denmark	59 ± 9	Asymptomatic T2DM patient Without cardiac disease	Cross-sectional/200	Oral antidiabetic medication (170), Insulin (124), RAAS blockade (196), Statin (189), Aspirin (183), Beta-blocker (27), Calcium channel blockers (80), Use of diuretics (128)	Plasma > 36.65	OR 2.54 [1.01–6.37], *p* = 0.048	Diabetes duration, vibration threshold, retinopathy and systolic blood pressure in the big toe	OPG was associated with CCS > 400 and coronary artery disease	CCS-CAD	Good
Ahmed [32]	Egypt	51.7	45 healthy subjects and 45 subjects with T2DM Free of CVD symptoms	Case–control/90	NR	Serum 12.9	OR 0.043 [0.006–0.296], *p* > 0.001	Age, gender and other risk factors	The CACS > 10 showed a significantly positive correlation with OPG	CACS	Moderate

**Table 2:** Correlation between OPG and traditional CVD risk factors

Study	Variable	Correlation coefficient	*P*-value
Anand [15]	Age	0.3	< 0.0001
	Waist-to-hip ratio	0.11	0.01
	Systolic blood pressure	0.11	0.01
	Duration of diabetes	0.2	< 0.0001
	BMI	− 0.04	0.35
	Diastolic blood pressure	0.03	0.57
	Smoking	0.07	0.11
	Total cholesterol	0.02	0.70
	LDL cholesterol	0.01	0.93
	HDL cholesterol	0.06	0.23
	Triglycerides	− 0.06	0.17
	Hemoglobin A1c	0.01	0.94
	Log hs-CRP	0.01	0.87
	Log IL-6	0.01	0.87
	Framingham risk score	0.016	0.0002
Ishiyama [16]	Mean intima-media thickness	r = 0.249	0.0011
Jung [31]	Age	0.373	0.002
	Pulse wave velocity	0.282	0.031
	BMI	0.015	0.920
	Fast blood sugar	0.140	0.251
	Hemoglobin A1c	0.077	0.535
	Total cholesterol	− 0.053	0.666
	HDL cholesterol	− 0.019	0.875
	LDL cholesterol	− 0.166	0.351
	Triglycerides	0.031	0.805
	Systolic blood pressure	0.142	0.271
	Diastolic blood pressure	0.035	0.787
	Duration of diabetes	0.065	0.635
	Creatinine	− 0.137	0.266
	hs-CRP	− 0.230	0.075
	CAC	0.336	0.005
Maser [28]	Age	0.22	> 0.05
	Gender	0.4	< 0.01
	Duration of diabetes	0.06	> 0.05
	Systolic blood pressure	0.25	> 0.05
	Diastolic blood pressure	− 0.02	> 0.05
	BMI	− 0.07	> 0.05
	Hemoglobin A1c	0.02	> 0.05
	Total adiponectin	0.30	> 0.05
	25-hydroxyvitamin D	0.09	> 0.05
	Parathyroid hormone	0.17	> 0.05
	Creatinine	− 0.24	> 0.05
	Leptin	0.3	< 0.05
	Calcium	0.33	< 0.05
Reinhard [29]	Age	0.26	< 0.001
	Duration of diabetes	0.18	0.010
	P-NT proBNP	0.27	< 0.001
	Heart rate variation	− 0.20	0.007
	CCS	0.24	0.001
Ahmed [32]	Age	0.2	0.001
	Fast blood sugar	0.3	0.001
	Duration of diabetes	0.9	0.001

OPG, osteoprotegerin; CVD, cardiovascular disease; BMI, body mass index; LDL, low-density lipoprotein; HDL, high-density lipoproteins; hs-CRP, high-sensitivity C-reactive protein; IL-6, interlukin-6; CAC, coronary artery calcification; CCS, coronary calcium score; CAD, coronary artery disease

### Association of OPG with CAC among type 2 diabetic patients

Regarding the relevant studies, the majority of those claimed that OPG could be associated with CAC among type 2 diabetic patients, except for one study that demonstrated no statistically significant correlation between OPG level and abnormal coronary artery calcium score among 168 type 2 diabetic subjects with a mean age of 60.85 years old. The result demonstrated that the mean level of OPG in diabetic subjects was 1.45 ± 0.63 pmol/L [16]. As seen in the study of Anand et al., comparing the data between baseline and follow-up indicated that plasma OPG could be associated with the elevated level of CAC among 510 multi-ethnical asymptomatic type 2 diabetic patients. By multivariate logistic regression analysis of CAC with OPG with the median level of 6.82 pmol/L, the odds ratio of 2.84 [2.2–3.67] was reported. The adjusted variables in this study were age, gender, hypertension, ethnicity, duration of diabetes, and statin use [15]. Likewise, serum level of OPG was significantly associated with CAC and it was indicated that circulating OPG could identify the individuals with an enhanced risk of arterial calcification (OR 3.324 [1.321–8.359]). The patients with abnormal CAC score had a mean level of OPG as 5.5 ± 2 pmol/L [28]. Moreover, the significant positive correlation between OPG and coronary calcium score was illustrated in a cross-sectional study recruiting 200 asymptomatic subjects with T2M (r = 0.24, p = 0.001). Accordingly, the high plasma concentration of OPG (higher than 36.65 pmol/L) remained a significant predictor of coronary calcium score ≥ 400 after adjustment for duration of diabetes, retinopathy, vibration threshold, and systolic blood pressure in the big toe (OR 2.54 [1.01–6.37]) [29]. In addition, OPG differed significantly in a nested case–control among 24 diabetic subjects with a mean age of 67.4 years old between the group having high CAC levels versus low CAC levels. The patients with high CAC score had a mean level of OPG as 9.3 pmol/L and the mean level of OPG as 7.7 pmol/L was measured in subject having low CAC levels [30]. Furthermore, Jung et al. performed a univariate analysis among 110 asymptomatic type 2 diabetic patients and indicated a significant correlation between CAC, circulating OPG levels, and arterial stiffness. As indicated by the result, the mean level of OPG in patients having CAC ≤ 10 was 10.07 ± 3.63 pmol/L and individuals with CAC > 10 had the mean level of OPG as 12.5 ± 4.83 pmol/L [31]. As seen in a study comparing serum OPG concentration between 45 patients with diabetes and 45 healthy age, and sex-matched individuals, higher levels of OPG (the mean level of OPG in diabetic patients was 12.9 ± 5.7 pmol/L and the mean level of OPG in non-diabetic controls was 8.6 ± 0.5 pmol/L) showed a positive correlation with coronary artery calcium score, although the reported OR contradict their findings [32].

## Summary effect estimate for the included studies

**Fig. 3:**
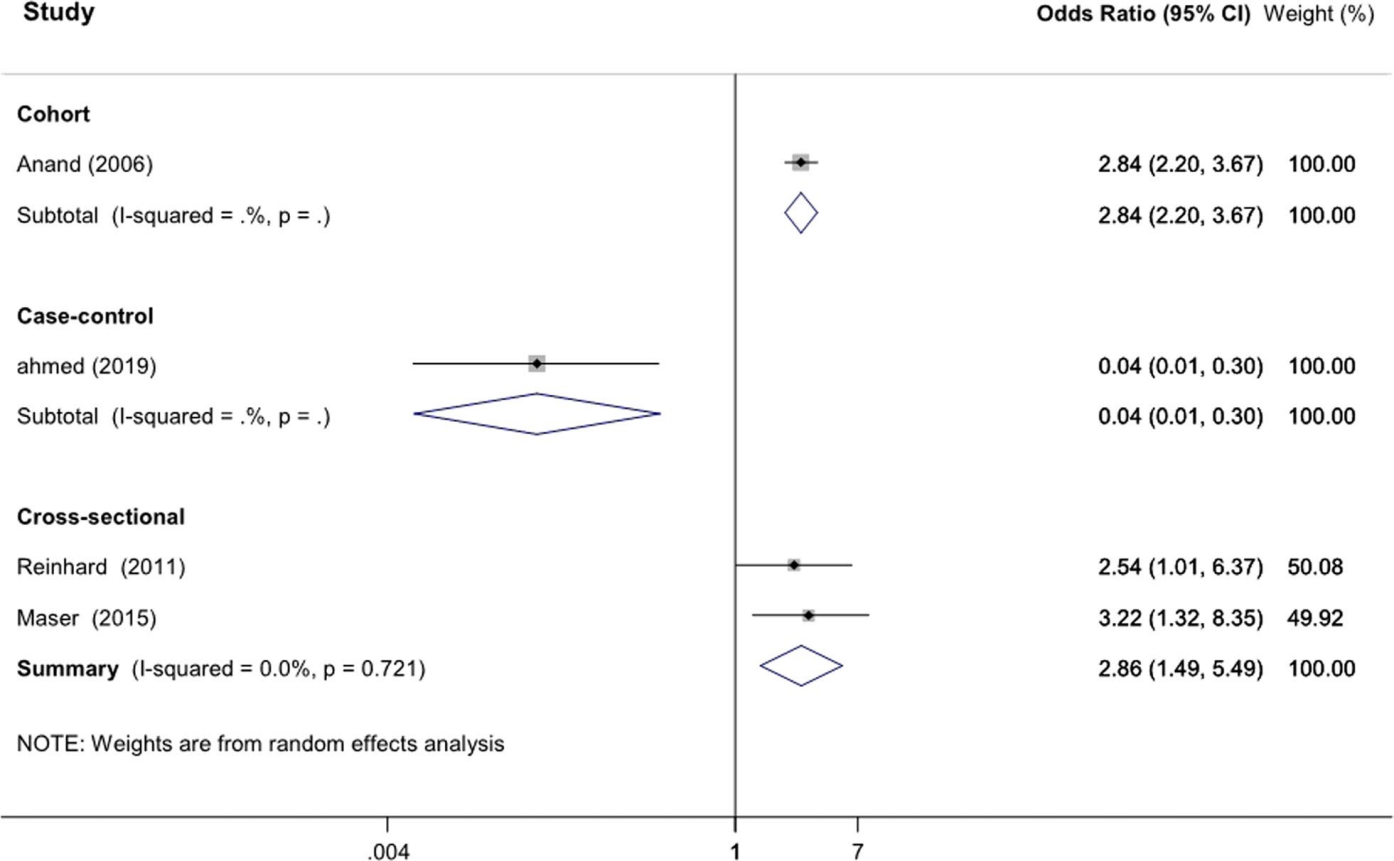
Visual summary of effect estimates of the studies. Pooled odds ratio of the results is presented based on the random effect model

Owing to the high heterogeneity in methodological and study design, meta-analysis could not be performed by this study. However, in order to visual inspection, pooled estimation of the results was presented in Fig. 3. After excluding of studies with incomplete data, four studies were eligible to be included in the summary effect estimate model. Regarding the cross-sectional studies investigating 250 type 2 diabetic patients without CVD symptoms, the pooled OR for CAC was estimated as 2.86 [95% CI 1.49–5.49] with relatively negligible heterogeneity between the studies (I^2^ = 0%).

## Discussion

Our systematic review found that elevated levels of OPG could possibly expose diabetic patients to a higher odds of abnormal coronary artery calcification scores more than 2.5 times. The result of the pooled OR for cross-sectional studies is consistent with the findings of the included cohort study. The abnormal coronary artery calcium score is regarded as an integrated factor indicating that individuals of different ages are prone to CVD burden among all races and genders. CAC testing is suggested to be done for predicting cardiovascular events and risk stratification by the recent American heart association guidelines, highlighting the value of CAC as a diagnostic marker in CVD [13, 33–36]. Increasing evidence in the clinical settings indicated that vascular calcification interferes with the surgeries required for revascularization among patients suffering from adverse CVD complications. For instance, after percutaneous coronary intervention (PCI) surgery it was seen that severe CAC can enhance the risk of ischemic events or even increase post PCI risks including delayed vascular healing, and stent under-expansion in patients [37–39]. Given the unfavorable impacts of CAC on cardiovascular treatment, suggesting a marker for predicting the presence of CAC that can assist in applying preventive approaches for vascular calcification would decrease cardiac mortality and morbidity. Therefore, OPG can play a prognostic role in determining CAC risk among subjects with T2M, that would be useful for identifying susceptible individuals to CAC burden in hyperglycemic conditions.

As seen in the prospective cohort study, diabetic patients were susceptible to a higher risk of CAC incidence following the elevated level of OPG, implying that high OPG levels were correlated with the duration of T2M [15]. Moreover, Reinhard et al. analyzed the association between OPG and CAC and the risk of CAD among asymptomatic type 2 diabetic subjects. It was reported that elevated levels of OPG could increase the risk of higher coronary calcium score by more than two times. In addition, multivariate analysis indicated that type 2 diabetic patients with a high level of OPG are exposed to the elevated incidence of CAD by about three folds independent of covariates, including age, sex, total plasma cholesterol, creatinine, peripheral systolic blood pressure, vibratory perception threshold, and heart rate variability [29]. Likewise, multiple regression analyses reported that subjects with elevated levels of serum OPG might be exposed to an increased risk of CAC by more than threefold; hence, OPG could be considered as a valuable marker for identifying type 2 diabetic individuals with the likelihood of arterial calcification incidence [28]. There is increasing evidence indicating the predictive role of OPG in CAD risk stratification among various populations. For instance, in a recent study that patients with acute myocardial infraction (AMI) were recruited, OPG was an independent prognostic marker for major adverse cardiovascular events (MACE) during one year follow-up and was also positively associated with AMI [40].

There are limited studies describing the mechanistic linkage between OPG and diabetes. Emerging evidence from in vitro studies demonstrated that vascular smooth muscle cells (VSMCs) would be transformed into osteoblast-like phenotype cells after being exposed to a hyperglycemic state, and these cells can facilitate the production of OPG in the vascular endothelial system [41, 42]. Likewise, high blood glucose could directly promote the expression of OPG in VSMCs and suppress RANKL production, suggesting that hyperglycemia could induce vascular calcification [11]. In addition, there is limited evidence from human clinical studies claiming that OPG is correlated with HbA1c. The multiple regression analyses in a study recruiting 26 type 1 diabetic children indicated that OPG levels could be significantly predicted by HbA1c [43]. As seen in our findings, the majority of studies reported that OPG could possibly be considered a prognostic marker for identifying abnormal CAC and early diagnosis of CVD incidence among type 2 diabetic populations. The pooled OR for the two cross-sectional studies with 250 type 2 diabetic patients was in favor of the result of the cohort study.

In agreement with the previous studies, Ahmed et al. [32] showed elevated OPG concentration in diabetic patients than controls. As they indicated a significant positive correlation between OPG and CAC, their findings were consistent with the results of the cohort and pooled cross-sectional studies.

Our results are consistent with previous studies demonstrating a significant association of high OPG concentrations with abnormal coronary artery calcium scores in asymptomatic subjects and patients with a history of CAD [44, 45]. As seen in a study recruiting 3386 subjects from the Dallas cohort population, asymptomatic individuals with an enhanced level of OPG had a higher risk of CAC incidence by 39% and were susceptible to the occurrence of vascular erosions and atherosclerosis [44]. Evaluating the association between OPG and CAC incidence in subjects with a history of CAD indicated the same results, and 45% of CAC prevalence was due to the elevated levels of OPG in these individuals [45].

The present systematic review has some limitations. OPG concentrations were measured in both plasma and serum in the studies. Hence, a calibration method for OPG measurement would report coordinate results. The observed various CAC cut-offs were another source of heterogeneity between different studies. Nevertheless, our results imply the association between OPG and two groups of high and low CAC score.

In addition, the existed gap of limited studies in this field merits further clinical evidence to recognize diabetic individuals at high risk of CVD. In case of quality assessment, robust reporting criteria, including those suggested by the EQUATOR (Enhancing the Quality and Transparency Of health Research) network, can be applied by authors to fortify data reporting [37]. The evidence obtained from seven studies, of which only one was a prospective cohort, showed pronounced variation in the aims and study design, which substantially restricted data pooling. Moreover, our analysis was based on observational studies that were susceptible to various biases. Hence, further primary studies especially prospective cohort studies with larger sample sizes with respect to the limitations of other studies could assist in verifying the association between OPG and coronary artery calcium score that lead to identifying diabetic populations that are susceptible to the elevated risk of cardiovascular events.

## Conclusions

OPG concentration can be proposed as a useful marker for identification of abnormal coronary artery calcium score among type 2 diabetic patients. However, the robust association of OPG and CAC needs to be further investigated by high quality longitudinal studies. Our findings in this review would provide clinical evidence for further studies of identifying OPG in predicting CAC burden in hyperglycemic conditions.

## Supplementary Information

**Additional file 1. Appendix 1**: PRISMA 2020 Checklist. **Appendix 2**: search strategy. **Appendix 3**: NOS assessment of the studies.

## Data Availability

The datasets generated during and/or analyzed during the current study are available from the corresponding author on reasonable request.
